# Fine-tuning and structured prompting strategies for question answering over full-text biomedical research articles

**DOI:** 10.1371/journal.pone.0351631

**Published:** 2026-06-24

**Authors:** Kaiming Tao, Rohit Satija, Jinru Zhou, Zachary A. Osman, Vineet Ahluwalia, Chiara Sabatti, Robert W. Shafer

**Affiliations:** 1 Division of Infectious Diseases, Dept. of Medicine, Stanford University, Stanford, California, United States of America; 2 Dept. of Biomedical Data Sciences, Stanford University, Stanford, California, United States of America; Government College Women University Sialkot: GC Women University Sialkot, PAKISTAN

## Abstract

**Objectives:**

The ability of large language models (LLMs) to answer targeted scientific questions by synthesizing information from research articles remains an open research challenge.

**Methods:**

We evaluated the effects of fine-tuning and a question-specific prompting strategy to answer 16 pre-defined questions about HIV drug resistance studies, including whether viral genetic sequences were reported and the demographics and antiviral treatments of the individuals from whom sequences were obtained. For fine-tuning, we constructed an instruction set comprising 250 HIV drug resistance studies, with 16 questions per study and corresponding answers and explanations. For question-specific prompting, we developed a set of if-then rules tailored to each question. We compared the performance of three base models – GPT-4o-mini-2024-07-18 (GPT-4o), Meta Llama-3.1-70B-Instruct (Llama-3.1-70B), and Meta Llama-3.1-8B-Instruct (Llama-3.1-8B) – with their performance using fine-tuning, question specific prompting, and fine-tuning followed by question-specific prompting. Performance was assessed using accuracy, precision, recall, and F1 score, averaged over 150 held-out studies not used for fine-tuning. Comparisons were performed using Wilcoxon signed-rank tests.

**Results:**

Fine-tuning increased precision by 5% for GPT-4o, 16% for Llama-3.1-70B, and 8% for Llama-3.1-8B, although this increase reached statistical significance only for Llama-3.1-70B. Fine-tuning also significantly increased recall for GPT-4o by 11%. Question specific prompting increased recall for all three models (6% for GPT-4o, 7% for Llama-3.1-70B, and 18% for Llama-3.1-8B), with statistically significant improvements observed only for Llama-3.1-8B. Applying question specific prompting to each of the fine-tuned models did not yield additional improvements beyond fine-tuning alone. When pooled across the three models, fine-tuning was associated with a greater effect on precision than recall (OR = 4.35; p = 0.001; Fisher’s exact test), whereas question-specific prompting led to a greater effect on recall than on precision (OR= 7.09; p = 0.0001; Fisher’s exact test).

**Conclusions:**

In this domain-focused proof-of-concept study, fine-tuning and question-specific prompting each led to improvement in one or more metrics for each of the three models. Pooled analyses indicated that fine-tuning improved precision, whereas question specific prompting preferentially improved recall.

## Introduction

The systematic review of data from multiple research studies is often required to answer many biomedical questions. The use of automated software tools to assist in reviewing research papers was a topic of interest that preceded the widespread use of large language models (LLMs) [1–7]. In the past three years, an increasing number of research studies have described the use of LLMs to screen papers for specific criteria, for summarizing their content, and for extracting specific data [8–20]. These investigations have largely focused on evaluating model performance in classifying publications based on abstracts or full texts. Several studies have also demonstrated the capacity of LLMs to extract targeted information from scientific articles [9,11,13,17–19].

We previously assessed the use of GPT-4 to answer questions about studies on HIV drug resistance [21]. In that study, we found that GPT-4 reproducibly answered a set of 60 questions with a precision of 87% and a recall of 73%. However, its performance was not improved with a 2000-word instruction sheet. The lack of improvement with this form of prompting, led us to assess the degree to which fine-tuning and a more targeted question-specific form of prompting could improve the performance of an LLM at answering specific questions about published research studies on HIV drug resistance.

Fine-tuning entails the continued training of a pre-trained LLM on domain-specific material for the purpose of adapting the model to specific tasks [22]. This process generally involves gradient-based updates to a subset of model parameters [23]. One of the most common approaches is instruction fine-tuning, in which instruction/ response pairs are employed to train the model on domain-specific tasks [24,25]. The datasets used for instruction fine-tuning may be created either through manual curation or via automated extraction using general-purpose LLMs [26,27]. A recent scoping review encompassing 37 studies reported that none employed fine-tuning to optimize model performance within a specific biomedical subdomain [28]. However, more recent accounts suggest an emergent interest in applying fine-tuning methods to support data abstraction during the conduct of systematic reviews [29,30].

We chose the topic of HIV drug resistance because we have extensive experience reviewing published studies on this topic as part of our work maintaining the Stanford HIV Drug Resistance Database and have published multiple systematic literature reviews on the topic [31–34]. We selected questions designed to determine whether a study reported HIV sequences and whether the sequences and their associated data were made publicly available. A fine-tuned model or advanced prompting strategy capable of answering questions about viral sequences, their public availability, and the demographics and antiviral treatments of the persons from whom the sequenced viruses were obtained would be invaluable to virology researchers, journal editors, and funding agencies.

## Materials and methods

### Research papers

For fine-tuning, we selected 250 curated research papers about HIV drug resistance from the Stanford HIV Drug Resistance Database encompassing studies of (1) HIV sequences from infected persons who were either antiretroviral treatment-experienced or treatment-naïve; (2) HIV isolates with known mutations undergoing *in vitro* susceptibility testing; and (3) different approaches to HIV sequencing and cloning. The complete list of papers is provided in the S1 File.

For testing, we assembled a test set comprising 150 research papers. The test set included 130 studies identified by querying PubMed for journal articles on HIV drug resistance published between 2023 and 2025. We then added 20 additional studies from the Stanford HIV Drug Resistance Database that reported data on uncommon topics unlikely to be represented in the first PubMed-derived set. As with the fine-tuning set, these papers included studies of viral sequences from HIV-infected persons, *in vitro* susceptibility testing, and technical aspects of HIV sequencing. S2 File lists the 150 papers used for testing.

For both fine-tuning and testing, a python script was used to convert each paper into a standardized markdown format containing the study title, abstract, methods, results, discussion, figure legends, and data-sharing statement.

### Research questions

We designed 16 questions addressing key aspects of HIV drug resistance including: (1) whether sequencing was performed on HIV isolates obtained from patients and whether resulting sequences were made publicly available (5 questions); (2) the demographics of patients whose viruses were sequenced (2 questions); (3) the treatment characteristics of patients whose viruses were sequenced (5 questions); and (4) technical aspects of sequencing (4 questions). Eight questions required list-based responses, seven required binary (yes/no) response, and one required a numeric response. For studies in which sequencing was not performed, answers to questions on patient demographics, treatments, and technical aspects of sequencing were considered to be “not reported”. Table 1 presents the complete list of questions along with the frequency with which answers were classified as true (for Boolean questions), non-empty (for list-based questions), or non-zero (for the numeric question) in both the 250-study instruction set and the 150-study test set.

**Table 1:** Complete List of Questions with their Frequencies of True, Non-Empty or Non-Zero in Both Instruction Set and Test Set.

	Question	Subject	Type	Instruction set (%)	Test set (%)
Q1	Does the paper report HIV sequences from patient samples?	Data availability	Boolean	85.6	68.0
Q2	Does the paper report in vitro drug susceptibility data?	Data availability	Boolean	20	18.0
Q3	Were sequences from the paper made publicly available?	Data availability	Boolean	56.4	18.7
Q4	What were the GenBank accession numbers for sequenced HIV isolates?	Data availability	List	54.4	14.7
Q5	How many individuals had samples obtained for HIV sequencing?	Data availability	Number	82.8	66.7
Q6	From which countries were the sequenced samples obtained?	Demographics	List	76.8	60.0
Q7	From what years were the sequenced samples obtained?	Demographics	List	64	57.3
Q8	Were samples cloned prior to sequencing?	Technical	Boolean	2.8	6.0
Q9	Which HIV genes were reported to have been sequenced?	Technical	List	91.2	83.3
Q10	What method was used for sequencing?	Technical	List	64.8	54.0
Q11	What type of samples were sequenced?	Technical	List	79.2	61.3
Q12	Were any sequences obtained from individuals with virological failure on a treatment regimen?	Treatment	Boolean	36.4	41.3
Q13	Were the patients in the study in a clinical trial?	Treatment	Boolean	14.4	14.0
Q14	Does the paper report HIV sequences from individuals who had previously received ARV drugs?	Treatment	Boolean	46.4	49.3
Q15	Which drug classes were received by individuals in the study before sample sequencing?	Treatment	List	36.8	44.7
Q16	Which drugs were received by individuals in the study before sample sequencing?	Treatment	List	34.4	38.0

### Fine-tuning

**Fig 1:**
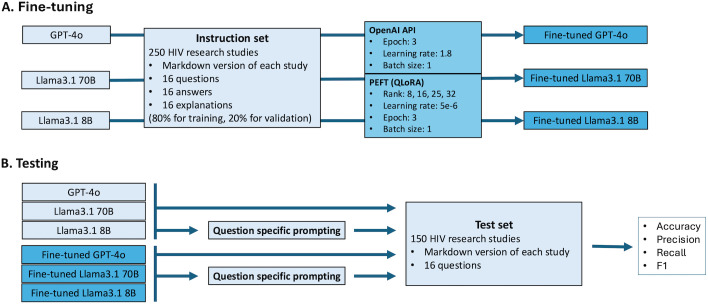
Approach to fine-tuning (FT), question-specific prompting (QSP), testing, and comparative analyses performed in this study. Fine-tuning was performed using an instruction set comprising 250 markdown versions of research studies, 16 questions about each study, and corresponding answers and explanations. GPT-4o was fine-tuned using the OpenAI API; Llama3.1-70B and Llama3.1-8B were fine-tuned using QLoRA (A). The performance of three base models (GPT-4o, Llama-3.1-70B, and Llama-3.1-8B) with their performance using fine-tuning, question-specific prompting, and fine-tuning followed by question-specific prompting. Performance was assessed using accuracy, precision, recall, and F1 score, averaged over 150 held-out test studies not used for fine-tuning. Comparisons between models were performed using Wilcoxon signed-rank tests with p-values adjusted using the Benjamini–Hochberg procedure to control the false discovery rate at 5% (B).

The instruction set comprised 250 training samples. Each sample contained (1) a markdown version of one of the 250 papers containing its title, abstract, methods, results, discussion, figure legends, and data sharing statement; (2) the 16 research questions; (3) the answers to each question; and (4) an explanation for each answer, including the text relevant to that answer (Fig 1A). For questions not addressed by a study, the explanation indicated that the study did not address the question. The complete training set is provided in the S3 File.

We used Hugging Face’s parameter efficient fine-tuning framework [35] using Quantized Low-Rank Adaptation (QLoRA) [23,36]. Given the complexity of the task and the length of the input samples, we selected a LoRA rank of 25 for Llama-3.1-70B and Llama-3.1-8B, which lies toward the upper end of the commonly recommended range of 4–32. As a sensitivity analysis, we also examined the effect of using three other ranks for the two Llama models – 8, 16, and 32. The OpenAI API did not provide options for rank. We set the batch size to one because the training samples were large, with a median of 5,503 tokens per sample (range: 1,091−17,458).

Table 2 summarizes the GPU, VRAM, and time requirements associated with fine-tuning and testing each model used in this study. For GPT-4o-mini-2024-07-18 (GPT-4o), the GPU and VRAM requirements were not available because fine-tuning and testing were performed using the OpenAI API [37].

**Table 2:** GPU, VRAM, and Time Requirements Associated with Fine-Tuning and Testing.

	Fine-Tuning			Testing the Base Model			Testing the Fine-Tuned Model		
Model	GPU	VRAM	Time	GPU	VRAM	Time	GPU	VRAM	Time
GPT-4o-mini	NA	NA	2h	NA	NA	1h	NA	NA	1h
Llama3.1 8B	1 A100	80G	1h	1 A100	80G	2h	2 A100	160G	13h
Llama3.1 70B	3 A100	240G	7h	3 A100	240G	5h	4 A100	320G	21h

Footnote: GPU (graphical processing unit); VRAM (video random access memory); A100 (Nvidia A100 tensor core GPU); VRAM is indicated as gigabytes.

### Question-specific prompting

We reviewed the fine-tuning instruction set and, for each question, extracted a series of if–then rules designed to guide an LLM on how to answer questions based on key phrases and higher-level conceptual cues. These rules were consolidated into a single prompt, with items ordered according to the sequence in which questions were presented to each LLM. The prompt was developed using an approach analogous to a recently published chain-of-thought method for improving automated screening in systematic literature reviews [38]. The complete prompt is provided in the S4 File. When tested, placing the prompt before the full text of a paper yielded results equivalent to placing it after, or both before and after, the paper; therefore, we positioned it before the paper in all evaluations.

### Retrieval-augmented generation (RAG)

Because each model was provided with the complete text of a research paper, RAG was not expected to substantially improve performance, as it is typically used to extend an LLM’s effective knowledge base or to enable querying of corpora that exceed the model’s context window by retrieving relevant text fragments at inference time. Nonetheless, given its widespread use, we implemented a retrieval-based approach. Specifically, we used the OpenAI text-embedding-3-small model to generate embeddings for section-aware text chunks (1,800 characters with one-paragraph overlap) from each of the 150 test papers [39]. For each of the 16 questions per paper, cosine similarity search was used to retrieve the top five most relevant chunks. Retrieved chunks were de-duplicated and were provided in place of the full-text article in prompts to the three base models.

### Testing and analysis

Fig 1B outlines the approach to testing used in this study. We evaluated three LLMs: (1) GPT-4o; (2) Meta Llama-3.1-70B-Instruct (Llama-3.1-70B; 70B parameters); and (3) Meta Llama-3.1-8B-Instruct (Llama-3.1-8B; 8B parameters). For each model, we compared four configurations: the base model, the fine-tuned model, question-specific prompting of the base model, and question-specific prompting of the fine-tuned model. For each question, model-generated responses were compared to the human-curated ground truth answers.

For the seven Boolean questions, we calculated the number of true positives, true negatives, false positives, and false negatives, as well as the model’s precision, recall, accuracy, and F1-score. For the eight list-based questions, we defined a true positive when at least two-thirds of the items in the human and model lists matched. A result was considered false negative when fewer than two-thirds of the human-list items were present in the model list. A result was considered false positive when >50% more items appeared in the model list than in the human list. For the sole numeric question, a result of 0 was considered analogous to an empty list. The S5 File lists the correct answers and the answers for each of the models for 2400 questions (150 papers x 16 questions).

We used the Wilcoxon signed-rank test method to compare the base model with the fine-tuned model, the base model with question-specific prompting, and the base model with fine-tuning followed by question-specific prompting. This was done separately for each of the three models. For each question, accuracy, precision, recall, and F1 score were calculated as the mean performance across 150 test studies. In accompanying figures illustrating these comparisons, each bar shows model-level performance computed from pooled true positive, true negative, false positive, and false negative counts across the full evaluation set. The Benjamini-Hochberg procedure was applied to control the false-discovery rate (FDR) at 5% across the three comparisons, three models, and four metrics.

Therefore, model performance was primarily compared at the level of the 16 predefined questions. For each question, accuracy, precision, recall, and F1 scores were calculated by aggregating results across all 150 papers, and paired comparisons between approaches were performed using the Wilcoxon signed-rank test. This approach treats each question as an independent unit of analysis, thereby accounting for variability in question difficulty and avoiding inflation of statistical significance that could arise from treating each paper–question prediction as an independent observation.

However, for analyses examining performance on individual questions, where outcomes consisted of paired binary predictions for each paper, we used McNemar’s tests to compare the proportion of discordant pairs (i.e., instances in which one model was correct and the other was incorrect). Because McNemar’s test applies to metrics defined at the level of individual predictions (e.g., accuracy and recall), it cannot be directly applied to precision, which is conditioned on predicted positives. The Benjamini-Hochberg procedure was applied to control the FDR at 5% across the three models and two strategies (fine-tuning and question-specific prompting).

### Human subjects research

This study did not involve human participants, human data, or human tissue. The research was based entirely on the evaluation of LLMs’ ability to extract information from previously published studies. As such, ethical approval and informed consent were not required.

## Results

### Comparison of the baseline models

**Fig 2:**
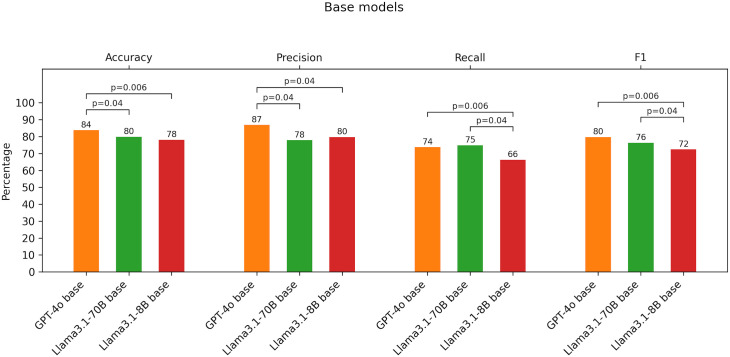
Comparison of the base models across the 16 evaluation questions. For each question, accuracy, precision, recall, and F1 score were calculated as the mean performance across 150 test studies. Statistical comparisons between models were performed using Wilcoxon signed-rank tests on the 16 paired question-level mean values, with p-values adjusted for multiple comparisons using the Benjamini–Hochberg procedure to control the false discovery rate at 5%. The raw data including the 95% confidence intervals for each bar are shown in the S6 File.

Fig 2 compares the overall mean accuracy, precision, recall, and F1 score for each base model across the 16 questions, pooled over the 150 test studies. Prior to fine-tuning, GPT-4o demonstrated significantly higher accuracy and precision than Llama-3.1-70B, and significantly higher accuracy, precision, and F1 score than Llama-3.1-8B, based on Wilcoxon signed rank tests adjusted for multiple comparisons. Under the same conditions, Llama-3.1-70B demonstrated significantly higher recall and F1 score than Llama-3.1-8B.

### Effect of fine-tuning, question-specific prompting, and their combination

**Fig 3:**
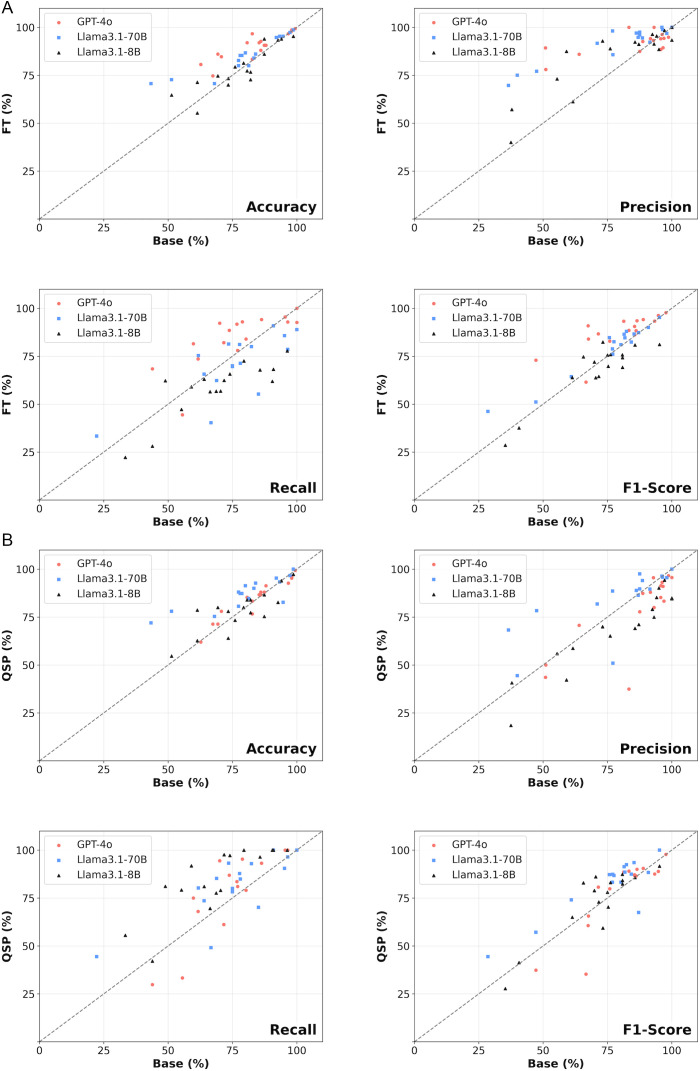
Impact of fine-tuning (FT; A) and question-specific prompting (QSP; B) on model performance across 16 evaluation questions applied to 150 test studies. Separate panels show accuracy, precision, recall, and F1 score. Each point represents the mean performance for a single question and a single model, averaged across the 150 test studies. The performance of the base model is shown on the X-axis, while the performance after fine-tuning (A) or with question-specific prompting applied to the base model (B) is shown on the Y-axis. Points above the diagonal line indicate questions for which fine-tuning or question-specific prompting improved performance relative to the base model, whereas points below the diagonal indicate decreased performance.

Fig 3A displays the accuracy, precision, recall, and F1-score of the base model and fine-tuning for each of the 16 questions for each of the three models. Fig 3B displays the corresponding comparisons between the base models and models using question-specific prompting. In both panels, points above the diagonal line indicate questions for which performance improved relative to the base model.

Across all three models and questions (48 comparisons), fine-tuning was associated with improved precision in 34 of 48 cases, but with improved recall in only 17 of 48 cases. In contrast, question-specific prompting was associated with improved recall in 35 of 48 cases, but with improved precision in only 13 of 48 cases. Consistent with these patterns, fine-tuning had a significantly greater effect on precision than on recall (OR = 4.35; p = 0.001; Fisher’s exact test) whereas question-specific prompting had a greater effect on recall than on precision (OR= 7.09; p = 0.0001; Fisher’s exact test).

**Fig 4:**
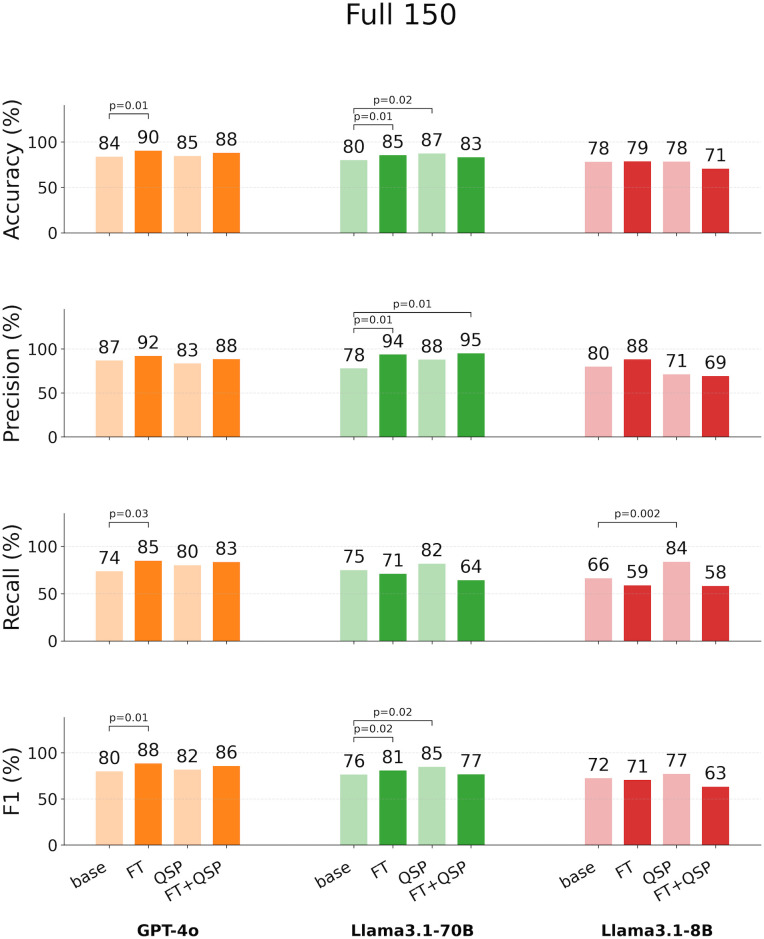
Effects of fine-tuning (FT) and question-specific prompting (QSP) on model performance. Effects of fine-tuning, question-specific prompting, and question-specific prompting applied to the fine-tuned model on accuracy, precision, recall, and F1 score for GPT-4o, Llama-3.1-70B, and Llama-3.1-8B. For each comparison, Wilcoxon signed-rank tests were applied to the 16 paired question-level mean values, with p-values adjusted for multiple comparisons using the Benjamini–Hochberg procedure to control the false discovery rate at 5%. The raw data including the 95% confidence intervals for each bar are shown in the S6 File.

Fig 4 summarizes the effects of fine-tuning, question-specific prompting and question-specific prompting applied to the fine-tuned model on accuracy, precision, recall, and F1 score for GPT-4o, Llama-3.1-70B, and Llama-3.1-8B across the 16 questions, pooled over the 150 test studies. For each model and metric, performance under fine-tuning, question-specific prompting, and fine-tuning followed by question-specific prompting was compared with the corresponding base model using Wilcoxon signed-rank tests adjusted for multiple comparisons.

Across all three models, fine-tuning increased precision by 5% for GPT-4o, 16% for Llama-3.1-70B, and 8% for Llama-3.1-8B; however, this increase reached statistical significance only for Llama-3.1-70B. Fine-tuning also significantly increased recall for GPT-4o. In addition, fine-tuning significantly improved both accuracy and F1-score for GPT-4o and Llama-3.1-70B.

Question-specific prompting increased recall by 6% for GPT-4o, 7% for Llama-3.1-70B, and 18% for Llama-3.1-8B, with statistically significant improvements observed for Llama-3.1-8B. Question-specific prompting significantly improved accuracy and F1-score for Llama-3.1-70B but not for the other models. Applying question-specific prompting to the fine-tuned models did not yield additional improvements beyond fine-tuning alone for any of the three models.

### Comparison of models after fine-tuning and question-specific prompting

**Fig 5:**
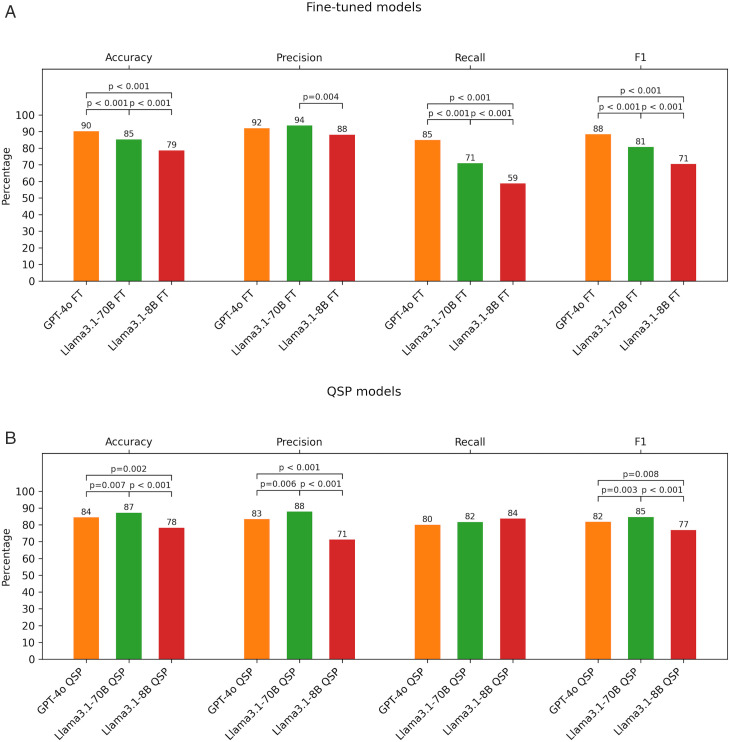
Comparison of models after fine-tuning (FT; A) or question-specific prompting (QSP; B). For each question, accuracy, precision, recall, and F1 score were calculated as the mean performance across 150 test studies. Statistical comparisons between models were performed using Wilcoxon signed-rank tests on the 16 paired question-level mean values, with p-values adjusted for multiple comparisons using the Benjamini–Hochberg procedure to control the false discovery rate at 5%. The raw data including the 95% confidence intervals for each bar are shown in the S6 File.

Fig 5A compares model performance after fine-tuning. Among fine-tuned models, GPT-4o achieved significantly higher accuracy, recall, and F1-score than both Llama-3.1-70B and Llama-3.1-8B, while precision did not differ significantly between GPT-4o and Llama-3.1-70B. Across all four metrics, Llama-3.1-70B significantly outperformed Llama-3.1-8B.

Fig 5B compares model performance using question-specific prompting. Under this strategy, Llama-3.1-70B achieved significantly higher accuracy, precision, and F1-score than GPT-4o, although the absolute magnitude of the differences was modest (3% to 5%). GPT-4o, in turn, significantly outperformed Llama-3.1-8B on accuracy, precision, and F1-score, with no significant difference in recall.

### Effect of fine-tuning and question-specific prompting on individual questions

Table 3 summarizes the research questions for which fine-tuning or question-specific prompting resulted in a statistically significant improvement in either accuracy or recall for each model. All comparisons were assessed using McNemar’s test for paired outcomes, with adjustment for multiple comparisons to account for the evaluation of two optimization strategies across three models.

**Table 3:** Accuracy and Recall for Questions With an Improvement After Fine-Tuning (FT) or Question-Specific Prompting (QSP).

	Question	Accuracy			Recall		
		B	FT	QSP	B	FT	QSP
***GPT-4o***							
2	Does the paper report in vitro drug susceptibility data?	82.7	96.7^**^	76.7	100	92.6	100
6	From which countries were the sequenced samples obtained?	80.7	92.0^**^	85.3	70.0	92.2^**^	94.4^**^
7	From what years were the sequenced samples obtained?	86.0	92.7	88.0	78.8	92.9^**^	95.3^**^
9	Which HIV genes were reported to have been sequenced?	66.7	74.7	71.3	60.8	73.6^**^	68.0
11	What type of samples were sequenced?	70.7	84.7^**^	78.0	59.8	81.5^**^	75.0^**^
12	Were any sequences obtained from individuals with virological failure on a treatment regimen?	88.0	90.7	91.3	73.8	88.5^*^	86.9
14	Does the paper report HIV sequences from individuals who had previously received ARV drugs?	87.3	90.7	88.0	76.7	91.8^*^	83.6
15	Which drug classes were received by individuals in the study before sample sequencing?	68.7	86.0^**^	71.3	70.1	82.1	61.2
16	Which drugs were received by individuals in the study before sample sequencing?	62.7	80.7^**^	62.0	43.9	68.4^**^	29.8
***Llama3.1-70B***							
1	Does the paper report HIV sequences from patient samples?	77.3	82.7	88.0^*^	73.5	81.4	93.1^**^
6	From which countries were the sequenced samples obtained?	80.0	86.7	91.3^**^	77.8	81.1	87.8^*^
7	From what years were the sequenced samples obtained?	84.0	86.0	92.7^*^	82.4	80.0	92.9^*^
9	Which HIV genes were reported to have been sequenced?	66.7	70.7	75.3	62.4	65.6	73.6^*^
10	What method was used for sequencing?	78.7	85.3	87.3	61.7	75.3	80.2^*^
12	Were any sequences obtained from individuals with virological failure on a treatment regimen?	83.3	84.0	90.0	68.9	62.3	85.2^*^
15	Which drug classes were received by individuals in the study before sample sequencing?	51.3	72.7^**^	78.0^**^	85.1	55.2	70.1
16	Which drugs were received by individuals in the study before sample sequencing?	43.3	70.7^**^	72.0^**^	66.7	40.4	49.1
***Llama3.1-8B***							
1	Does the paper report HIV sequences from patient samples?	82.0	76.7	77.3	79.4	72.5	100^**^
5	How many individuals had samples obtained for HIV sequencing?	73.3	73.3	78.0	64.0	63.0	81.0^*^
6	From which countries were the sequenced samples obtained?	69.3	74.7	80.0	48.9	62.2	81.1^**^
7	From what years were the sequenced samples obtained?	80.7	77.3	84.0	71.8	62.4	97.6^**^
9	Which HIV genes were reported to have been sequenced?	60.0	55.3	78.7^**^	53.6	47.2	79.2^**^
12	Were any sequences obtained from individuals with virological failure on a treatment regimen?	79.3	81.3	80.0	59.0	59.0	91.8^**^
14	Does the paper report HIV sequences from individuals who had previously received ARV drugs?	76.0	79.3	73.3	74.0	65.8	97.3^**^
16	Which drugs were received by individuals in the study before sample sequencing?	51.3	64.7^*^	54.7	43.9	28.1	42.1

Footnote: Abbreviations: B (base model). **McNemar’s test adjusted p < 0.01; *McNemar’s test adjusted p < 0.05.

The most pronounced effect was that question-specific prompting significantly increased recall for six questions for Llama-3.1-70B and seven questions for Llama-3.1-8B. In contrast, for GPT-4o, an increase in recall was driven primarily by fine-tuning which led to improvements in seven questions including three questions for which question-specific prompting also led to significant improvements.

A similar but less pronounced pattern was observed for accuracy. Fine-tuning led to significant improvements in accuracy for five questions for GPT-4o. In contrast, for Llama-3.1-70B, question-specific prompting led to improvements for five questions including two for which fine-tuning also led to significant improvements. For Llama-3.1-8B, fine-tuning and question-specific prompting each improved accuracy for one question.

Several questions (questions 6, 7, 9, 12, and 16) demonstrated improvements in either accuracy or recall for all three models. For example, for question 9 (“Which HIV genes were reported to have been sequenced?”), question-specific prompting significantly improved recall for both Llama-3.1-70B and Llama-3.1-8B, whereas fine-tuning significantly improved recall for GPT-4o.

### Question-level performance and error analysis

**Fig 6:**
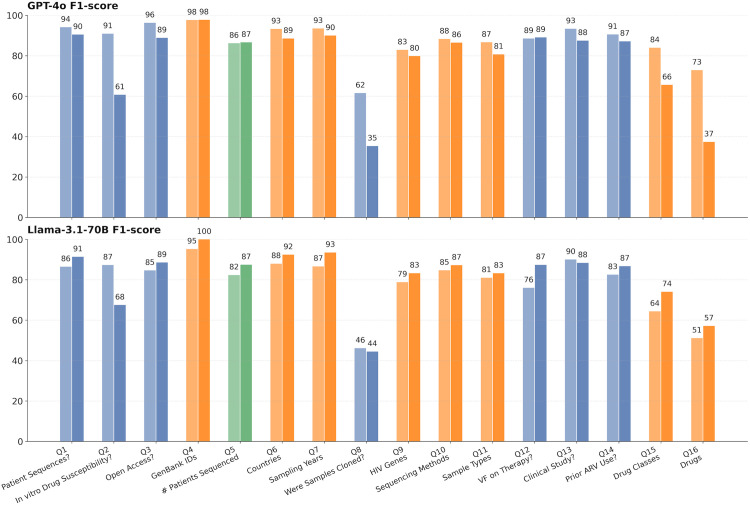
Question level performance and error analysis. F1-scores for GPT-4o (top panel) and Llama-3.1-70B (bottom panel) across the 16 study questions, stratified by question type: Boolean (blue), list-based (orange), and numeric (green). For each question, bars on the left show performance after fine-tuning, and bars on the right show performance after question-specific prompting. The raw data including the 95% confidence intervals for each bar are shown in the S6 File.

To identify systematic patterns in model performance, we examined question-level F1-scores for the two best-performing models (GPT-4o and Llama-3.1-70B) under fine-tuning and question-specific prompting (Fig 6). The lowest F1-scores were observed for questions related to cloning (Q8) and antiretroviral therapy, including drug classes and individual drugs (Q15 and Q16), indicating persistent difficulty with these question types across both models.

Across the 16 questions, there were five instances in which performance differed by more than 10 percentage points between fine-tuning and question-specific prompting. For the question assessing whether a study reported *in vitro* drug susceptibility data (Q2), both GPT-4o and Llama-3.1-70B achieved substantially higher F1-scores following fine-tuning than following question-specific prompting. In addition, for each of the three questions with the lowest overall F1-scores, GPT-4o showed markedly higher performance after fine-tuning than after question-specific prompting, suggesting that fine-tuning mitigated some of the largest failure modes observed with rule-based prompting alone.

To better characterize the sources of question-level errors observed consistently for the two best-performing models under both fine-tuning and question-specific prompting, we conducted a qualitative review of representative failure cases (Table 4). These examples show that models frequently struggled to identify narrowly defined analytic subsets when sequencing or genotyping was described only indirectly, often defaulting instead to larger cohort-level denominators. Additional errors reflected conflation of related but distinct concepts, such as interpreting resistance prevalence as evidence of prior drug exposure or inferring gene sequencing solely from the reporting of mutations. Beyond the error types illustrated in Table 4, we also observed cases in which the relevant information was presented exclusively in tables, figure legends, or embedded text rather than stated explicitly in the main text.

**Table 4:** Illustrative cases of extraction failures by GPT-4o and Llama-3.1-70B under fine-tuning (FT) and question-specific prompting (QSP).

Question	Relevant Text from Study	Model Error Summary
How many individuals had samples obtained for HIV sequencing?	“PDVF … was observed in 11/112 at W24 and 14/112 at W48.” (PMID: 36659824).	The correct answer was 14. FT answered Not reported due to missing explicit sequencing language; QSP outputs returned 112 by anchoring on the study denominator rather than the PDVF (protocol-defined virological failure) subset.
	“Out of 33 samples eligible for genotyping…” (PMID: 37993493).	The correct answer was 33. Llama-3.1-70B FT failed to extract the explicit count; GPT-4o FT and both QSP outputs responded 600, the number of persons undergoing viral-load testing (reported elsewhere in the paper) with the genotyped subset.
	“GRTs were able to evaluate drug resistance … only in five patients.” (PMID: 38090027).	The correct answer was 5. Most FT/QSP outputs missed the embedded subset and answered *Not reported*; one QSP output returned *1040,* anchoring on the cohort size.
Which HIV genes were reported to have been sequenced?	“Six and four had new reverse transcriptase and integrase mutations…” (PMID: 35945163).	The correct answer was reverse transcriptase (RT) and integrase. All FT/QSP outputs answered *Not reported*, reflecting conservative extraction when mutations are mentioned without explicit gene-sequencing statements.
	“All 51 participants … including the four with Met184Val …” (PMID: 37541705).	The correct answer was RT. All FT/QSP outputs answered *Not reported*, presumably due to absence of any sequencing description, even though an RT-associated mutation (Met184Val) was mentioned.
	“GRTs … none selected variants resistant to integrase inhibitors…” (PMID: 38090027).	The correct answer was RT and integrase. All FT/QSP outputs answered *Not reported*; inferring RT and integrase would require domain conventions not stated in text.
Which drugs were received by individuals before sample sequencing?	“… the most frequent high-level, medium-level, and low-level resistant drugs were NVP, ETR, and ABC …” (PMID: 37946329).	The correct answer was Not reported. All models inferred drug receipt (*NVP, ETR, and ABC*) from test results, conflating resistance outcomes with treatment exposure.
	“Treatment-naïve perinatally HIV-infected infant whose mother was receiving TDF/3TC/DTG.” (PMID: 37755428).	The correct answer was None. All models mistakenly attributed maternal ART to the infant.
	“10 (6%) met virological failure and were included in the RAP (TG1, n = 3; TG2, n = 2; TG3, n = 4; TG4, n = 1)…” (PMID: 41056006).	The correct answer was TAF, FTC, lenacapavir, and bictegravir. The RAP (resistance analysis plan) subset and TGs (treatment groups) were described earlier in the paper. One model extrapolated *lenacapavir* from treatment-group descriptions; others answered *Not reported*, reflecting difficulty linking acronyms to regimen descriptions.

Footnote: Abbreviations: PMID – PubMed ID; GRT – genotypic resistance test; NGS – next-generation sequencing; RT – reverse transcriptase; NVP – nevirapine; ETR – etravirine; ABC – abacavir; TDF – tenofovir; 3TC – lamivudine; DTG – dolutegravir; ART – antiretroviral therapy.

### Effect of RAG and study length on model performance

Application of RAG to the 150 study test set resulted in slightly higher recall compared with baseline for GPT-4o, Llama-3.1-70B, and Llama-3.1-8B. This difference reached statistical significance only for Llama-3.1-8B. However, RAG displayed lower recall than question-specific prompting across each model and RAG displayed lower accuracy, precision, and F1 scores compared with the full text baseline for each model (S8 File).

**Fig 7:**
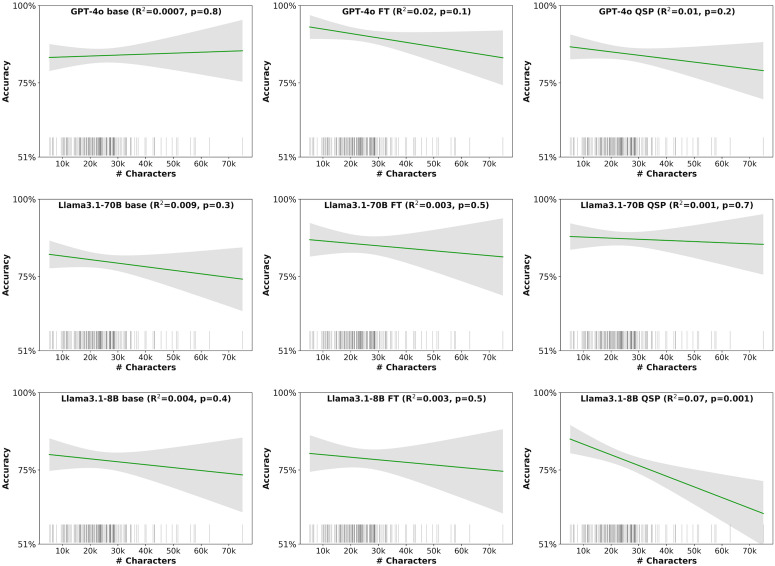
Relationship between document length and model accuracy. Accuracy is plotted as a function of document length (x-axis), measured as the total number of characters in each full-text article. The green line represents the fitted regression trend for each model and condition. Shaded areas indicate the 95% confidence interval around the fitted trend line. Tick marks along the x-axis denote the distribution of individual observations (articles) included in the analysis.

To assess whether study length influenced model performance, we examined the relationship between accuracy and the number of characters (including spaces) in each of the 150 test studies for each model and inference strategy (base model, fine-tuning, and question-specific prompting; Fig 7). The median study length was approximately 23,000 characters, corresponding to roughly 5,800 tokens. Across all but one model–strategy combination, there was no significant association between study length and accuracy (R² range: 0.0005–0.01). The only exception was Llama-3.1-8B with question-specific prompting, which exhibited a modest inverse association between study length and accuracy (R² = 0.067; p = 0.01). Inspection of the data indicates that this association was driven primarily by a small number of unusually long studies (>50,000 characters), with minimal variation in accuracy across the majority of shorter studies.

### Effect of the size of the instruction set on the performance of fine-tuning

**Fig 8:**
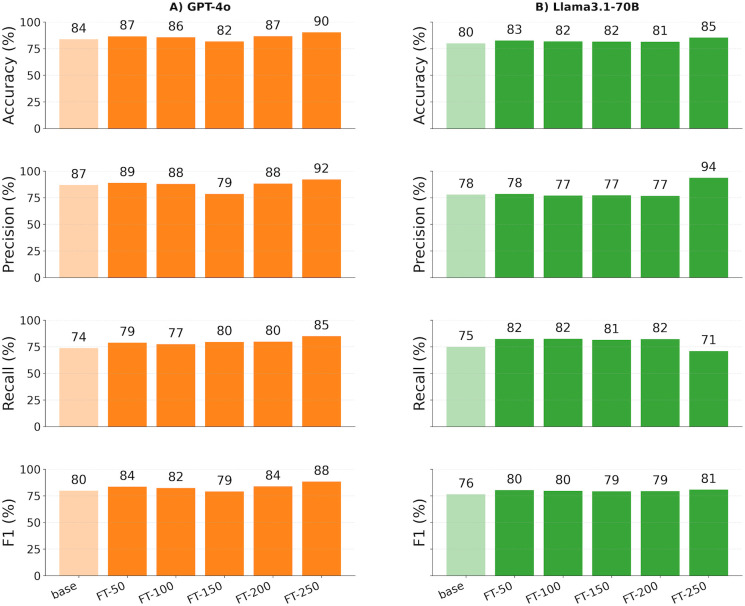
Fine-tuning learning curves as a function of instruction set size. Effect of instruction set size on fine-tuning performance for GPT-4o (A) and Llama-3.1-70B (B). Accuracy, precision, recall, and F1 score were evaluated on 150 held-out test studies comprising 16 questions per study. For each question, performance was calculated as the mean across the 150 test studies, yielding one value per question. Bars show the average of these per-question mean values for the base model (Base) and after fine-tuning using random subsets of 50, 100, 150, 200, or all 250 studies from the full instruction set. The raw data including the 95% confidence intervals for each bar are shown in the S6 File.

To assess how the size of the instruction set used for fine-tuning influenced model performance, we performed learning-curve analyses evaluating accuracy, precision, recall, and F1 score for GPT-4o and Llama-3.1-70B. For each model, we generated four random subsets of the original 250-paper instruction set containing 50, 100, 150, and 200 studies, and fine-tuned the models using the same hyperparameters applied to the full 250-paper instruction set. Fig 8A and 8B present the results for GPT-4o and Llama-3.1-70B, respectively.

Across seven of the eight model–metric combinations (two models × four metrics), performance increased in an approximately monotonic manner as the size of the instruction set increased. The sole exception was recall for Llama-3.1-70B, which showed a marked improvement even with the smallest instruction subset, followed by an unexplained decrease when fine-tuned on the complete instruction set.

### Effect of QLoRA rank on the performance of fine-tuned Llama-3.1-70B and Llama-3.1-8B models

S9 File shows the effect of using lower (8, 16) or higher (32) QLoRA ranks relative to the rank of 25 used in the primary analyses. Using the Cochran-Armitage Test for Trend, we found that recall for Llama-3.1-70B decreased significantly and monotonically with increasing rank (p < 0.001). In contrast, no significant differences in recall were observed across ranks for Llama-3.1-8B.

## Discussion

Answering pre-specified questions about scientific research papers remains a difficult and largely unsolved problem. Unlike simpler information-extraction tasks, the answers to many research questions are not stated directly in a single sentence or paragraph. Instead, they often require understanding domain-specific terminology, combining information spread across different sections of a paper, and applying background knowledge that authors assume but do not explicitly describe [16,18,28,40,41].

In this study, we assessed the degree to which fine-tuning and a question-specific form of prompting could improve the performance of LLMs at answering specific clinically and epidemiologically relevant questions about studies in a narrow biomedical domain. We examined the baseline performance of three LLMs with their performance after fine-tuning, question-specific prompting, or fine-tuning followed by question-specific prompting.

Our main findings were that fine-tuning increased precision by 5% for GPT-4o, 16% for Llama-3.1-70B, and 8% for Llama-3.1-8B, although after adjustment for multiple comparisons this increase reached statistical significance only for Llama-3.1-70B. Fine-tuning also significantly increased recall for GPT-4o by 11%. Question specific prompting increased recall for all three models (6% for GPT-4o, 7% for Llama-3.1-70B, and 18% for Llama-3.1-8B), with statistically significant improvements observed for Llama-3.1-8B. When pooled across models, fine-tuning was associated with a strong significantly greater effect on precision than recall, whereas question-specific prompting led to a greater effect on recall than on precision. Applying question-specific prompting to the fine-tuned models did not yield additional improvements beyond fine-tuning alone.

The differential effects of fine-tuning and question-specific prompting on precision and recall suggest a possible trade-off between these strategies. Fine-tuning may constrain model outputs toward patterns learned from curated training data, thereby reducing spurious responses and improving precision. In contrast, question-specific prompting may encourage broader exploration of potentially relevant evidence, increasing sensitivity at the expense of additional false positives. This pattern is consistent with prior studies comparing fine-tuning and prompting-based approaches for complex question-answering tasks [42,43].

An additional finding of this study was that fine-tuning was associated with statistically significant improvements in accuracy and F1 score only for GPT-4o and Llama-3.1-70B but not for Llama-3.1-8B. This observation is consistent with prior studies showing that parameter-efficient fine-tuning methods such as LoRA and QLoRA yield larger gains in higher-capacity models. Smaller models appear to be more strongly constrained by representational capacity, such that low-rank adapters provide limited additional benefit, particularly for tasks requiring long-context reasoning and synthesis across multiple document sections [23,26,30,36].

Our qualitative error analysis further suggests that many errors reflect limitations of the source literature. Even after fine-tuning or question-specific prompting, models struggled when key information was implicit, dispersed across sections, or dependent on domain-specific conventions that were not explicitly stated. These findings underscore that accurate question answering over full-text research articles often requires resolving ambiguity about analytic scope and conceptual intent, challenges that are not fully addressed by improved pattern matching alone.

Most prior studies evaluating the use of LLMs to answer questions about research studies have focused on determining whether a study’s title and abstract—and less frequently its full text—meet inclusion criteria for systematic reviews [8–15,28,30,38]. In contrast, we evaluated model performance using the full text of each study because the information required to answer our predefined questions was typically not reported in abstracts alone. For this reason, the instruction set developed for fine-tuning also included the full text of each paper. Although prior work has leveraged full-text articles for pretraining or to improve performance on general question-answering tasks, our study placed greater emphasis on repeated, question-specific supervised fine-tuning at the document level to assess how such an approach affects a model’s ability to answer the same set of domain-specific questions across research studies.

### Limitations

There are several key limitations to our study. First, we focused on a narrowly defined scientific domain, HIV drug resistance, within which there exists a large and methodologically heterogeneous body of published research. We also limited our questions to those with Boolean, list-based, or numeric answers, enabling objective evaluation of a large number of responses in the test set (n = 2400), rather than more interpretive questions such as clinical outcomes or methodological assessments. As noted in the Introduction, our choices were motivated by the practical demands of maintaining the Stanford HIV Drug Resistance Database, which requires accurate extraction and synthesis of information distributed across sections of full-text articles. Consequently, the specific fine-tuned models and question-specific prompting strategies described here are unlikely to be directly transferable to unrelated research domains. Rather, our findings demonstrate that, within a well-defined domain where accurate answers require integrating information distributed across multiple sections of a manuscript, both fine-tuning and question-specific prompting can meaningfully improve model performance.

Second, we did not perform sensitivity analyses for any fine-tuning hyperparameter other than rank. This study was intentionally designed as a pilot project to evaluate the feasibility and qualitative effects of fine-tuning and question-specific prompting for the concrete operational task of supporting curation of the Stanford HIV Drug Resistance Database rather than to optimize model performance. Accordingly, we adopted a fixed, practically motivated fine-tuning configuration, employing a QLoRA adapter rank of 25, training for three epochs with a batch size of one, and selecting learning rates using standard practices appropriate to each fine-tuning framework. A comprehensive sensitivity analysis would have substantially expanded the computational scope of the study and would have shifted its focus away from evaluating the relative behavior of fine-tuning and prompting strategies in a real-world curation setting. For future work aimed at broader generalization or deployment across additional domains, more extensive hyperparameter tuning could be explored.

Third, we focused on fine-tuning and prompting and did not extensively evaluate RAG and its variants. RAG is commonly used to extend an LLM’s effective knowledge base or to enable querying of corpora that exceed the model’s context window by retrieving relevant text fragments at inference time [44–46]. In our study, however, each model was provided with the complete text of each article, which averaged approximately 6,000 tokens and was well within the context limits of all evaluated models. Consistent with this, we observed no meaningful association between article length and model performance.

Nonetheless, we implemented a simple RAG-based approach in which complete documents were replaced with retrieved text fragments. This approach resulted in numerically lower accuracy, precision, and F1 scores compared with the full-text baseline across models, although recall increased for the smallest model. However, even in this case, performance remained inferior to that achieved with question-specific prompting. These findings suggest that, in settings where full-text inputs are available, retrieval-based approaches primarily alter evidence selection rather than expand available context and may not improve overall performance.

Fourth, some degree of prior exposure of base models to earlier HIV drug resistance publications cannot be completely excluded. However, answering our predefined questions required extracting and synthesizing highly specific methodological and contextual details that were often distributed across multiple sections of full-text articles and were not explicitly stated. Consistent with this, the base models frequently produced incorrect or incomplete answers, even for studies that may have been included in pretraining corpora, indicating that prior exposure alone was insufficient to reliably support accurate question answering.

Fifth, our approach to fine-tuning relied on a large, manually constructed instruction set and is therefore difficult to scale. An important future direction of our work is the development of more scalable training strategies that directly target common sources of misinterpretation. One potential alternative is the creation of focused training examples that pair short, error-prone text passages with explicit clarifications of their intended meaning.

## Conclusions and future directions

In this study, we showed that both fine-tuning and question-specific prompting can meaningfully improve the ability of LLMs to answer clinically and epidemiologically relevant questions from full-text biomedical research articles. Fine-tuning was more likely to improve precision, whereas question-specific prompting was more likely to improve recall. Performance gains were consistently larger for GPT-4o and Llama-3.1-70B than for the smaller Llama-3.1-8B model. Taken together, these findings suggest a practical trade-off between precision and recall that may be useful when choosing between approaches.

Despite ongoing advances in LLMs, some degree of task-specific adaptation – through fine-tuning, structured prompting, or both – will likely remain necessary to achieve performance comparable to, or exceeding, that of human data extraction. Although human curation is itself imperfect, with prior studies reporting error rates of 10–30% even with duplicate abstraction for complex or poorly reported data elements [47–49], these limitations underscore the value of continued efforts to improve LLM-based extraction.

Our error analysis also highlighted the extent to which limitations in reporting practices contribute to model failures. This observation reinforces the importance of clearer, more explicit reporting and other efforts to improve the machine-readability of biomedical publications [50,51]. Improvements in both model capabilities and reporting practices will be necessary to realize the full potential of automated extraction systems. This would also facilitate future work on data types of relevance across studies of other pathogenic human viruses, including those with available antiviral therapies as well as those with pandemic potential.

## Supporting information

S1 File Research papers in the instruction set. (XLSX)

S2 File Research papers in the test set. (XLSX)

S3 File Instruction set used for fine tuning. (XLSX)

S4 File Question-specific prompts. (DOCX)

S5 File Human and model answers for test set questions. (XLSX)

S6 File Data for figures along with confidence intervals. (XLSX)

S7 File Statistical Summaries for Table 3. (XLSX)

S8 File RAG Comparison Figure. (DOCX)

S9 File QLoRA Sensitivity Analysis Figure. (DOCX)
