# Supplementary material for: Fine-tuning and structured prompting strategies for question answering over full-text biomedical research articles
Source: PLoS One. 2026 Jun 24;21(6):e0351631. doi: 10.1371/journal.pone.0351631 (PMC13293408; doi:10.1371/journal.pone.0351631)
Supplement: S4 File — (DOCX) [file pone.0351631.s004.docx]

**Study Objectives**

The objective of this project is to systematically extract and curate data related to HIV drug resistance from published reports. For each paper, identify and summarize information on:

1. HIV nucleotide or amino-acid sequences obtained from clinical samples, including GenBank accession numbers.
2. The populations from whom HIV sequences were obtained.
3. The clinical samples and sequencing methods used.
4. Antiretroviral (ARV) treatment histories of the individuals whose samples were sequenced.

**Question 1 – Does the paper report HIV sequences from patient samples?**

**Goal:** Determine whether the study generated new HIV nucleotide or amino-acid sequences, or reported lists of mutations, directly from clinical samples obtained from individuals with HIV (ART-naïve or ART-experienced).

**Answer “Yes” if the paper:**

- States that genetic sequencing, genotypic resistance testing, Sanger sequencing, next-generation sequencing, or single-genome sequencing was performed on clinical samples such as plasma, serum, PBMCs, proviral DNA, or dried blood spots.
- Indicates that sequencing was done on samples from patients, participants, subjects, blood donors, newly diagnosed or newly infected individuals, PLWH/PWH, or cohorts of individuals with HIV.
- States that surveillance for transmitted or acquired drug resistance was performed and generated new sequence data.
- Reports GenBank accession numbers for sequences generated in the study.

**Answer “No” if:**

- Only laboratory strains (e.g., HXB2, IIIB, NL4-3, BAL, LAI) or site-directed mutants were studied.
- The paper is a review or meta-analysis using sequences reported in other studies or downloaded from databases without generating new sequences.

**Question 2 – Does the paper report in vitro drug susceptibility data?**

**Goal:** Determine whether the paper quantifies HIV in vitro susceptibility to ARV drugs.

**Answer “Yes” if the paper:**

- States that phenotypic susceptibility testing, phenotypic resistance testing, or a drug susceptibility assay was performed.
- Reports IC₅₀, EC₅₀, EC₉₀, fold change in susceptibility, or similar measures.
- Describes a susceptibility assay (e.g., PhenoSense/Monogram, Antivirogram/Virco) or gives details on cells, drug titrations, and reporter readouts.
- Specifies the virus used for testing (e.g., clinical isolate, site-directed mutant, virus from in vitro passage).

Otherwise, answer “No”.

**Question 3 – Were sequences from the paper made publicly available?**

**Goal:** Determine whether newly generated HIV sequences or raw reads were deposited in public repositories (e.g., GenBank, SRA, DDBJ, ENA).

**Answer “Yes” if the paper:**

- Provides GenBank accession numbers (typically two letters followed by digits) in the Methods, Results, a Supplementary Table, or Data Availability statement.
- States that sequences have been submitted to GenBank or another public database, even if accession numbers are “pending.”
- States that raw reads are available in SRA or other sequence-read repositories.

**Answer “No” if:**

- Only accession numbers for HIV reference strains (e.g., HXB2, NL4-3, LAI, BAL) are given.
- No accession numbers or deposition statements are provided.
- Only accession numbers from previous studies are cited.
- The paper is a review or meta-analysis using existing sequence data.

**Question 4 – What are the GenBank accession numbers for sequenced HIV isolates?**

- If the paper reports that sequences were submitted to GenBank or another public database and provides accession numbers, extract all individual accession numbers and ranges (e.g., FJ800379–FJ800386; GQ477441–GQ477451; KP170487).
- Accession numbers may appear in the Methods, in a table, in Supplementary material, or in a Data Availability statement.
- If accession numbers are described but extraction is ambiguous, copy the relevant text describing the submission.

If no accession numbers are given, answer “Not reported”.

**Question 5 – How many individuals had samples obtained for HIV sequencing?**

- If the paper does **not** report HIV sequences from individuals with HIV, or only compiles sequences from databases/previous studies, answer **0**.
- If sequencing or genotypic resistance testing was performed on samples or sequences from X individuals, answer **X**.
- If the paper reports X isolates/sequences from a group of patients but does not specify how many individuals contributed isolates, assume one sequence per individual and answer **X**, unless stated otherwise.
- If multiple mutually exclusive patient groups are described, sum individuals across all groups.
- If sequences were generated but the number of individuals is not reported, answer **“Not reported”**.

**Question 6 – From which countries were the sequenced samples obtained?**

- If the paper does not report HIV sequences from individuals with HIV, answer **“Not applicable”**.
- Use the country or countries where samples were collected (e.g., recruitment, enrollment, follow-up, or surveillance site).
- If multiple countries are listed, report all.
- If samples were collected in country A and sequenced in country B, report **country A**.
- If no country information is provided, answer **“Not reported”**.

**Question 7 – From what years were the sequenced samples obtained?**

- If the paper does not report HIV sequences from individuals with HIV, answer **“Not applicable”**.
- If sample collection dates are reported (e.g., Year1–Year2, or month/year ranges), report the year span (e.g., “June 2004–April 2007” → **2004–2007**).
- If samples are listed by individual years (e.g., 2008, 2009, 2010), report the full span (e.g., **2008–2010**).
- If only months within a single year are reported (e.g., March–September 2008), report that single year (**2008**).
- If baseline sequencing corresponds to enrollment or diagnosis years, use that year range.
- If no sample-collection years are provided, answer **“Not reported”**.

**Question 8 – Were samples cloned prior to sequencing?**

- If the paper does not report HIV sequences from individuals with HIV **or** does not report in vitro susceptibility data, answer **“Not applicable”**.
- Answer **“Yes”** if:
  - The paper states that “amplicons were cloned and sequenced,” or describes use of a cloning method (e.g., TA/Topo TA cloning).
  - Molecular clones were sequenced.
  - Single-genome amplification or limiting dilution was performed prior to sequencing.
  - Site-directed mutagenesis was performed on isolates that were sequenced.
- Answer **“No”** if:
  - Direct PCR or population-based sequencing is reported.
  - A commercial genotyping platform (e.g., ViroSeq, TruGene, GeneSeqR, GenoSure, Vela Diagnostics) is used.
  - Next-generation sequencing (NGS) without cloning is used.
- If cloning information is not provided, answer **“Not reported”**.

**Question 9 – Which HIV genes were reported to have been sequenced?**

Report all applicable gene targets using the abbreviations **PR**, **RT**, **IN**, **Pol**, **CA** (capsid), and **Env**.

- If the paper states “pol genotyping/sequencing” without further detail, answer **“Pol”**.
- If “protease and reverse transcriptase,” “PR/RT,” “ViroSeq,” or “TruGene (PR/RT)” are mentioned, include **PR** and **RT**.
- If “integrase,” “IN,” “GeneSeq/ViroSeq Integrase,” “INSTI resistance,” or “integrase sequencing” are mentioned, include **IN**.
- If NRTI or NNRTI resistance is assessed, include **RT**.
- If PI resistance is assessed, include **PR**.
- If INSTI resistance is assessed, include **IN**.
- If lenacapavir resistance is assessed, include **CA** (capsid).
- If “env,” “gp120,” “gp41,” “gp160,” “V3 loop/C2V3,” or coreceptor-usage genotyping is reported, include **Env**.

**Question 10 – What method was used for sequencing?**

- Answer **“Sanger sequencing”** if:
  - Dideoxy (dideoxyterminator) or Sanger sequencing is explicitly mentioned.
  - ABI or Thermo Fisher sequencing reagents/instruments are used.
  - Capillary electrophoresis, electropherograms, or “direct PCR sequencing” are described.
  - The paper was published before 2008 (NGS was not widely used).
- Answer **“NGS”** if:
  - “Next-generation sequencing,” “NGS,” or “ultra-deep sequencing” is reported.
  - Illumina platforms (MiSeq, HiSeq, iSeq), Oxford Nanopore (e.g., GridION), PacBio RSII/SMRT, 454 pyrosequencing, Sentosa, or Vela NGS systems are used.
- If the sequencing method is not described, answer **“Not reported”**.

**Question 11 – What type of samples were sequenced?**

- If the paper does not report HIV sequences from individuals with HIV, answer **“Not applicable”**.
- If viral RNA was extracted from plasma or serum, include **“Plasma”**.
- If samples were obtained at diagnosis or at virological failure, they are likely plasma: include **“Plasma”**.
- If PBMCs, peripheral blood mononuclear cells, or proviral DNA are mentioned, include **“PBMC”**.
- If samples were from individuals with stable virological suppression, they are often PBMCs: include **“PBMC”**.
- If nucleic acids were extracted from whole blood, include **“Whole blood”**.
- If dried blood spots are used, include **“DBS”**.
- If other sample types (e.g., lymph node, cerebrospinal fluid) are used, list those specifically.
- If the specimen type for sequencing is not stated, answer **“Not reported”**.

**Question 12 – Were any sequences obtained from individuals with virological failure on a treatment regimen?**

- If the paper does not report HIV sequences from individuals with HIV, answer **“Not applicable”**.
- Answer **“Yes”** if:
  - Samples were from patients with virological failure or virological rebound.
  - Sequencing was performed on plasma from ART-experienced individuals.
  - Samples were obtained from patients on second-line treatment.
  - Patients had received NRTIs, NNRTIs, PIs, and/or INSTIs and sequences were generated.
- Answer **“No”** if:
  - All sequenced patients were newly diagnosed or ART-naïve.
  - Sequencing was done exclusively for transmitted drug-resistance surveillance (i.e., untreated individuals).

**Question 13 – Were the patients in the study in a clinical trial?**

- Answer **“Yes”** if the paper:
  - Describes randomization to different treatments.
  - Describes a study as double-blind, placebo-controlled, open-label, controlled, or multicenter.
  - Describes a phase 1, 2, or 3 trial.
- Answer **“No”** if the paper:
  - Describes an observational or prospective cohort, retrospective or cross-sectional study, case-control study, or case series.
  - Provides no information suggesting clinical-trial participation.

**Note:** Registration at ClinicalTrials.gov (NCT number) alone is not sufficient to classify a study as a clinical trial.

**Question 14 – Does the paper report HIV sequences from individuals who had previously received ARV drugs?**

- If the paper does not report HIV sequences from individuals with HIV, answer **“No”**.
- Answer **“Yes”** if sequences were obtained from individuals who:
  - Were receiving ART, were ART-experienced or treatment-experienced, or had virological failure/rebound.
  - Were receiving specific ARV classes (NRTIs, NNRTIs, PIs, INSTIs, capsid inhibitors, entry inhibitors) or specific drugs (e.g., 3TC, FTC, AZT, TDF, abacavir, efavirenz, rilpivirine, doravirine, atazanavir, lopinavir, darunavir, raltegravir, elvitegravir, dolutegravir, bictegravir, cabotegravir, lenacapavir, maraviroc, fostemsavir, enfuvirtide, ibalizumab).
  - Had stable virological suppression on ART.
  - Acquired HIV infection while receiving PrEP.
  - Include both ART-naïve and ART-experienced individuals (any history of ARV use → “Yes”).
- Answer **“No”** if **all** sequences were from ART-naïve, treatment-naïve, drug-naïve, previously unexposed, or newly diagnosed individuals, or from individuals in a pure transmitted drug-resistance surveillance study.

**Question 15 – Which drug classes were received by individuals in the study before sample sequencing?**

- If the paper does not report HIV sequences from individuals with HIV, answer **“None”**.
- If the paper does not report HIV sequences from individuals who had previously received ARVs, answer **“None”**.

Otherwise, list all applicable drug classes:

- If the paper names drug classes directly (NRTIs, NNRTIs, PIs, INSTIs, capsid inhibitors, entry inhibitors), list those.
- If only specific drugs are named, infer classes:
  - **NRTIs:** zidovudine/AZT, lamivudine/3TC, emtricitabine/FTC, tenofovir (TDF/TAF), abacavir/ABC, stavudine/d4T, didanosine/ddI.
  - **NNRTIs:** efavirenz/EFV, nevirapine/NVP, etravirine/ETR, rilpivirine/RPV, doravirine/DOR.
  - **PIs:** lopinavir/r (LPV/r), atazanavir/ATV, darunavir/DRV, saquinavir/SQV, indinavir/IDV, nelfinavir/NFV, tipranavir/TPV, fosamprenavir/FPV.
  - **INSTIs:** raltegravir/RAL, elvitegravir/EVG, dolutegravir/DTG, bictegravir/BIC, cabotegravir/CAB.
  - **Capsid inhibitors (CAIs):** lenacapavir/LEN.
  - **Entry inhibitors:** maraviroc/MVC, fostemsavir, enfuvirtide, ibalizumab.

Additional rules:

- If all sequences are from ART-naïve/treatment-naïve/drug-naïve individuals, answer **“None”**.
- If a pre-2020 paper states that individuals were receiving a first-line WHO regimen, include **NRTI** and **NNRTI**.
- If a pre-2020 paper states that individuals were receiving a second-line WHO regimen, include **NRTI**, **NNRTI**, and **PI**.
- If individuals are described only as “ART-experienced” without drugs or classes, answer **“Not reported”**.

**Question 16 – Which drugs were received by individuals in the study before sample sequencing?**

- If the paper does not report HIV sequences from individuals with HIV, answer **“None”**.
- If the paper does not report HIV sequences from individuals who had previously received ARVs, answer **“None”**.
- If sequences were obtained from ART-experienced individuals but no specific drugs are named, answer **“Not reported”**.
- If only drug classes (e.g., NRTIs, NNRTIs, PIs, INSTIs) are mentioned without individual drugs, answer **“Not reported”**.
- If specific ARV drugs are listed, report **all** drugs received (using generic names; abbreviations may be included in parentheses).
