# Supplementary material for: Fine-tuning and structured prompting strategies for question answering over full-text biomedical research articles
Source: PLoS One. 2026 Jun 24;21(6):e0351631. doi: 10.1371/journal.pone.0351631 (PMC13293408; doi:10.1371/journal.pone.0351631)
Supplement: S8 File — (DOCX) [file pone.0351631.s008.docx]

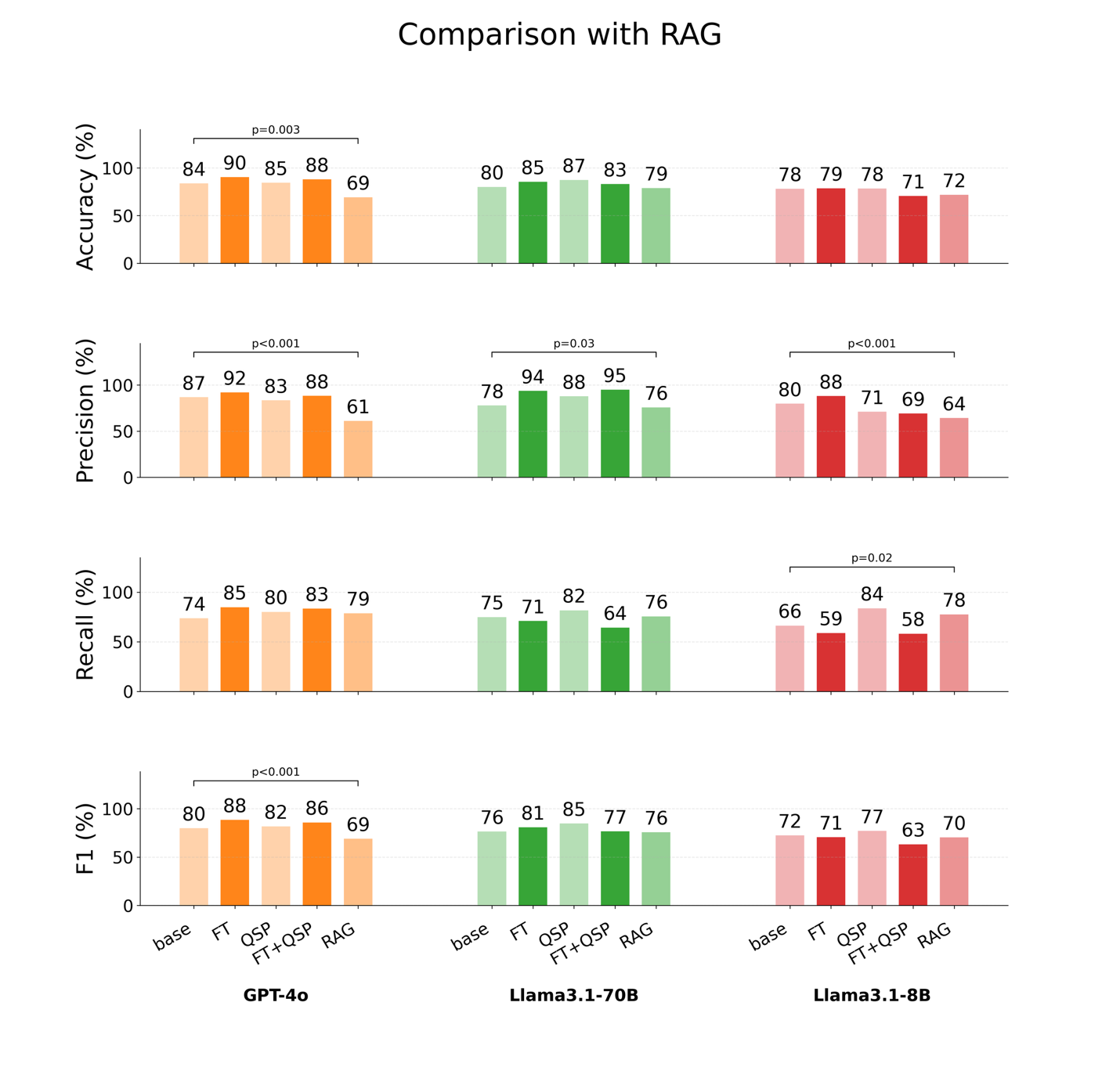


Supplementary Fig. Comparison with retrieval-augmented generation (RAG).

Accuracy, precision, recall, and F1 score are shown for GPT-4o, Llama-3.1-70B, and Llama-3.1-8B under base, fine-tuning (FT), question-specific prompting (QSP), FT+QSP, and RAG conditions. Bars show model-level performance computed from pooled TP, TN, FP, and FN counts across the full evaluation set. For RAG, the full-text article context was replaced by a shared, deduplicated evidence pool constructed from question-specific retrieval over the same paper. P-value annotations show Wilcoxon signed-rank tests comparing RAG with the corresponding full-text base model using paired question-level metric values across the 16 questions. P-values were adjusted for multiple comparisons using the Benjamini-Hochberg procedure to control the false discovery rate at 5%, across 48 Wilcoxon comparisons: 3 model families × 4 target conditions vs base (FT, FT+QSP, QSP, and RAG) × 4 metrics. The raw bar values and 95% confidence intervals are shown in S6 File.
