# Supplementary material for: Fine-tuning and structured prompting strategies for question answering over full-text biomedical research articles
Source: PLoS One. 2026 Jun 24;21(6):e0351631. doi: 10.1371/journal.pone.0351631 (PMC13293408; doi:10.1371/journal.pone.0351631)
Supplement: S9 File — (DOCX) [file pone.0351631.s009.docx]

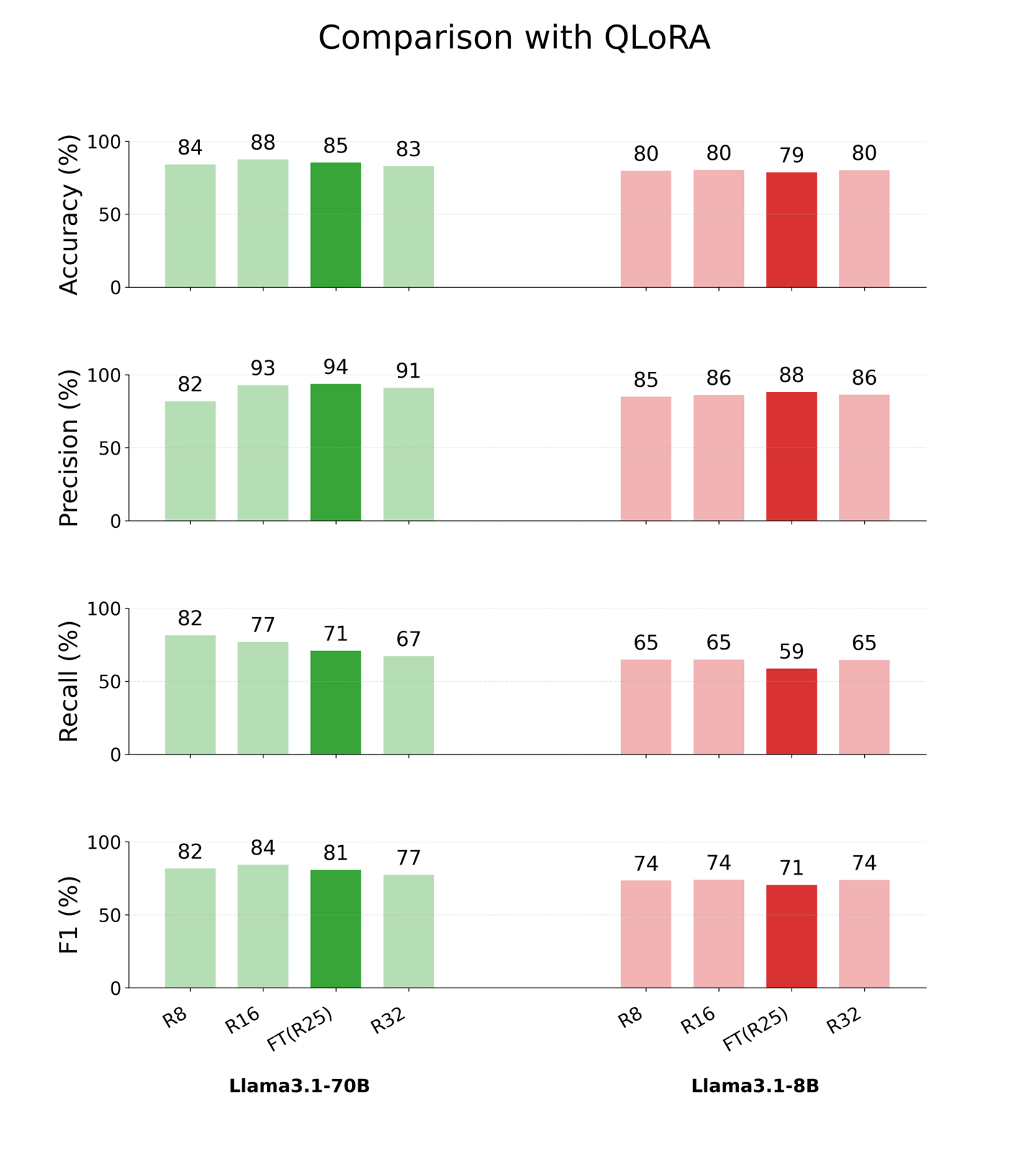


Supplementary Fig. Effects of quantized low-rank adaptation (QLoRA) at different ranks on model performance.
QLoRA with ranks of 8, 16, 25, and 32 were applied to the Llama-3.1-70B and Llama-3.1-8B models. Performance was evaluated using accuracy, precision, recall, and F1 score. Each bar shows model-level performance computed from pooled true positive, true negative, false positive, and false negative counts across the full evaluation set. For Llama-3.1-70B, performance decreased significantly and monotonically with increasing rank (p < 0.001). In contrast, no significant differences in recall were observed across ranks for Llama-3.1-8B (Cochran–Armitage test).
